# Retraction Note: Dandelion flower-fabricated Ag nanoparticles versus synthetic ones with characterization and determination of photocatalytic, antioxidant, antibacterial, and α-glucosidase inhibitory activities

**DOI:** 10.1038/s41598-026-54403-5

**Published:** 2026-05-26

**Authors:** Soheil Yousefzadeh-Valendeh, Mohammad Fattahi, Behvar Asghari, Zeinab Alizadeh

**Affiliations:** 1 Department of Horticulture, Faculty of Agriculture, Urmia University, Urmia, Iran; 2 Department of Horticultural Sciences Engineering, Faculty of Agriculture and Natural Resources, Imam Khomeini International University, Qazvin, Iran

Retraction of: *Scientific Reports* 10.1038/s41598-023-42756-0, published online 18 September 2023

The Editors have retracted this Article.

After publication, concerns were raised regarding the authenticity of the TEM data reported in the second and third images of Figure 3. These images appear to have been previously published as Figure 1a in^1^ and Figure 1b in^2^, respectively, where they correspond to different types of nanoparticles than the ones reported in this Article. The Authors were not able to provide the raw data underlying this figure. The Editors therefore no longer have confidence in the reliability of the data reported in this Article.

Mohammad Fattahi disagrees with the retraction. Soheil Yousefzadeh-Valendeh, Behvar Asghari, and Zeinab Alizadeh did not respond to the Editors’ correspondence about this retraction.
